# Atomic layer etching of graphene through controlled ion beam for graphene-based electronics

**DOI:** 10.1038/s41598-017-02430-8

**Published:** 2017-05-26

**Authors:** Ki Seok Kim, You Jin Ji, Yeonsig Nam, Ki Hyun Kim, Eric Singh, Jin Yong Lee, Geun Young Yeom

**Affiliations:** 1 School of Advanced Materials Science and Engineering, Sungkyunkwan University, 2066 Seobu-ro, Jangan-gu, Suwon-si, Gyeonggi-do 16419 Republic of Korea; 2 SKKU Advanced Institute of Nano Technology (SAINT), Sungkyunkwan University, 2066 Seobu-ro, Jangan-gu, Suwon-si, Gyeonggi-do 16419 Republic of Korea; 3 School of Chemistry, Sungkyunkwan University, 2066 Seobu-ro, Jangan-gu, Suwon-si, Gyeonggi-do 16419 Republic of Korea; 4 Department of Computer Science, Stanford University, Stanford, California 94305 United States

## Abstract

The electronic and optical properties of graphene are greatly dependent on the the number of layers. For the precise control of the graphene layers, atomic layer etching (ALE), a cyclic etching method achieved through chemical adsorption and physical desorption, can be the most powerful technique due to barely no damage and no contamination. In this study, we demonstrated the ALE process of graphene layers without noticeably damaging the graphene by using a controlled low energy oxygen (O_2_^+^/O^+^)-ion for chemical adsorption and a low energy Ar^+^-ion (11.2 eV) for physical desorption. In addition, using a trilayer graphene, mono- and bi-layer graphene could be successfully fabricated after one- and two-cycle ALE of the trilayer graphene, respectively. We believe that the ALE technique presented herein can be applicable to all layered materials such as graphene, black phosphorous and transition metal dichalcogenides which are important for next generation electronic devices.

## Introduction

Graphene has attracted considerable attention due to their unique properties including very high electron mobility, extremely high mechanical strength, superior thermal conductivity, and high chemical stability^1–4^. Especially, its electronic and optical properties greatly depend on the number of layers in graphene^5–9^. Accordingly, the accurate control of the number of layers is the most important technology in various of graphene-based device applications.

To control the layers of graphene, researchers have investigated various methods such as oxidation etching in a tube furnace^10^, Joule heating^11^, laser thinning^12, 13^, plasma etching using helum, nitrogen and oxygen^14–18^. However, these techniques can be difficult to achieve precise control of graphene layers at the atomic layer scale because the removal rate of graphene layers depends only on etch time. Also, for some methods, it is even difficult to remove the graphene layers without inducing damage and contamination to the surface.

Precise control of graphene layers by layer-by-layer removal has been reported by Dimiev *et al*.^19^ by the deposition of Zn on a photoresist patterned graphene surface, lift-off Zn on photoresist, and by the removal of patterned Zn on graphene surface with one graphene layer using HCl. Even though this method can remove graphene layers layer-by-layer precisely without damaging the graphene layer surface, it may not be applicable for nanoscale electronic device fabrication due to the requirement of a lift-off process used for patterning Zn on the graphene surface. Atomic layer etching (ALE), a cyclic etch method composed of chemical adsorption and physical desorption of the sequential steps, is also one of the techniques that have good potential in precisely removing graphene layer-by-layer without inducing damage and contamination. In addition, it is applicable for the nanoscale device fabrication because it removes graphene layers after photoresist patterning using coventional patterning methods.

In this research, layer-by-layer removal of graphene using ALE process using an ion beam has been investigated for the various next generation nanoscale graphene electronic devices. Using an optimized ALE process composed of a controllable low energy oxygen(O_2_^+^/O^+^)-ion for chemical adsorption and a low energy Ar^+^-ion beam for physical desorption, we were able to achieve a precise control of graphene layers without damage on the bottom graphene layer. Especially, for the photoresist patterned graphene or the chemical vapor deposited (CVD) graphene transferred from the Cu using Poly(methyl methacrylate)(PMMA), a thin polymer residue is remaining on the graphene surface even after the removal of the photoresist/PMMA. Using the low energy Ar^+^-ion, the polymer residue layer could be also completely removed in this study before the ALE process without damaging the graphene surface^20, 21^.

## Methods

### Preparation of layered graphene

Graphene was synthesized by a CVD method on Cu. After the graphene synthesis, the Cu foil was cut into pieces, coated with PMMA (Microchem 950 C), and immersed into a FeCl_3_ solution in order to etch away the Cu foil. When the Cu foil was completely removed, the graphene layer on PMMA was rinsed in deionized water to wash away etchant residues. Then, the PMMA-coated graphene layer was transferred onto a 300-nm-thick SiO_2_/Si wafer. PMMA on the SiO_2_/Si wafer was removed using acetone and a very thin PMMA residue remaining on the graphene surface was completely removed using a low energy Ar^+^-ion cleaning without damage to graphene bonding as described elsewhere^21^. For a bi- and tri-layer graphene, the above method was repeated two and three times, respectively, to obtain clean graphene layers on a 300-nm-thick SiO_2_/Si wafer.

### ALE equipment and process

**Figure 1:**
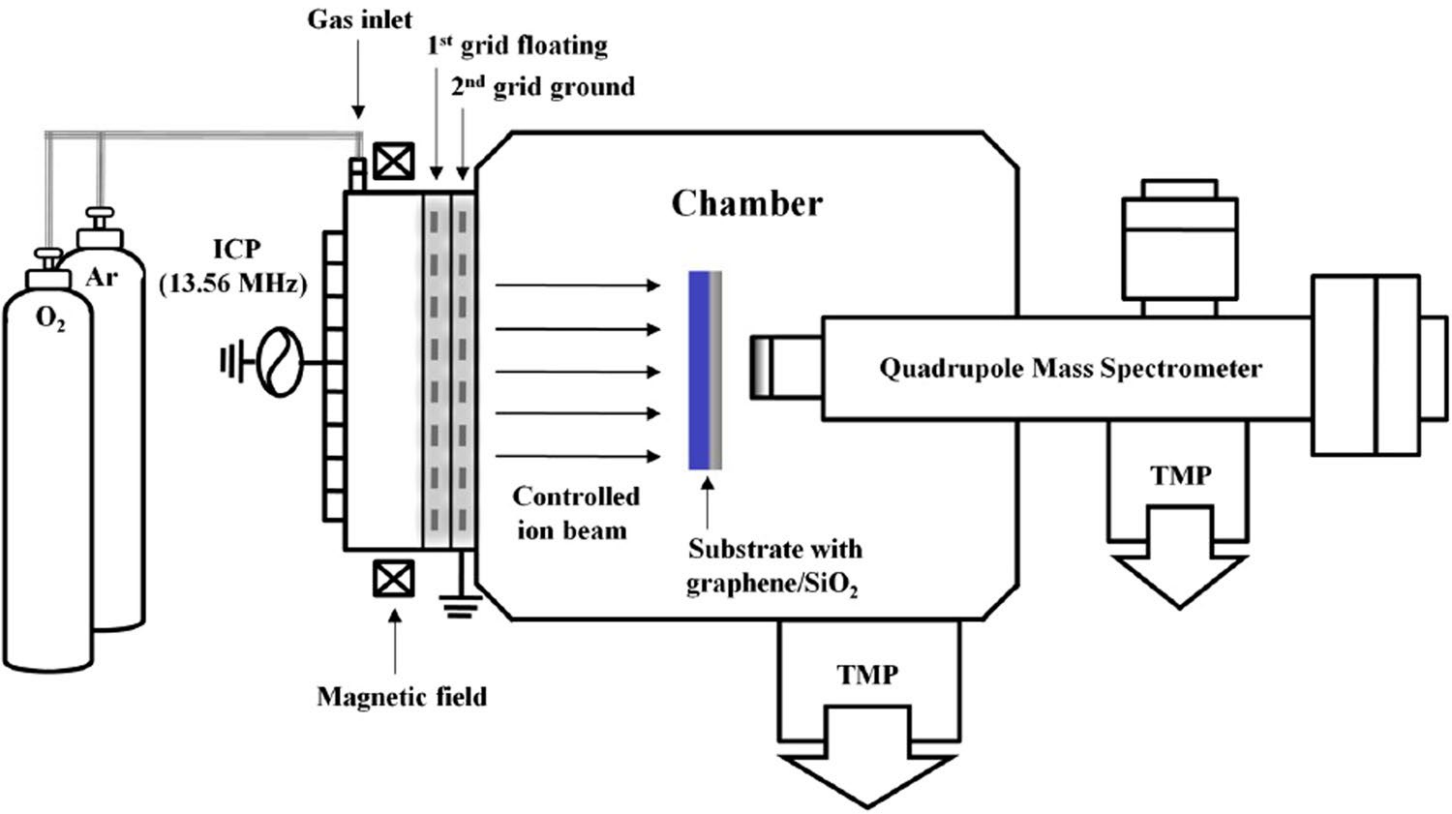
Schematic diagram. A two-grid ICP-type ion beam system with axial magnetic field used for graphene ALE and a quadrupole mass spectrometer for ion energy/flux measurement of the ion beam.

The schematic diagram of a two-grid (4inch-diameter) inductively coupled plasma (ICP)-type ion beam system with axial magnetic field used for the layer-by-layer removal of graphene, that is, for graphene ALE and a quadrupole mass spectrometer (QMS) installed for the measuremet of ion energy and flux of the ion beam is shown in Fig. 1.

To obtain a low energy oxygen(O_2_^+^/O^+^)-ion and a low energy Ar^+^-ion beam, the 1st grid of the ion beam source was floated without applying any voltage while the 2nd grid was grounded. The details of the ion beam system can be found elsewhere^21^. In some cases, an axial magnetic field of 30 Gauss was applied in front of the 1st grid to decrease the ion beam energy. To measure the ion energy distribution on the graphene surface distribution at various ion beam conditions such as different radio frequency (rf) powers and gas flow rates to the ion source and with/without axial magnetic field, a mass spectrometer was installed at the substrate location. As a result of this analysis, the following processes were used as the optimized ALE cyclic steps; for chemical adsorption step, an oxygen-ion beam generated with 100 sccm of O_2_, 15 W of rf power, and with axial magnetic field of 30 Gauss for 90 s and, for the physical desorption step, an Ar^+^-ion beam generated with 100 sccm of Ar and 500 W of rf power for 120 s.

### Simulation: graphene binding energy calculation

To investigate the graphene ALE mechanism by the chemical adsorption of oxygen and physical desorption by Ar ion bombardment, a computational simulation on the change of binding energies between carbon atoms on graphene before and after the oxygen adsorption was performed using Vienna Ab Initio Simulation Package (VASP)^22^. We performed density functional theory (DFT) calculation with a local-density approximations (LDA)^23^ and the projector-augmented wave (PAW)^24^ method. A 7 × 7 × 1 grid for *k*-point^25^ sampling and the energy cut-off of 400 eV were consistently used in our calculations. The convergence threshold for energy was set to 10^−5^ eV.

The lattice constant of graphene unit cell was calculated to be 2.45 Å after relaxation. Then, geometry optimization was carried out on the 3 × 3 × 1 supercell structure. To describe the bilayer graphene, two-dimensional periodic boundary conditions were used along the growth direction with vacuum space of at least 10 Å to avoid additional interaction between the layers. For the bilayer graphene structure, both A-A stacking and A-B stacking structure were considered (Figure S1, Supplementary Information).

The binding energy of an atom (E_B_) was calculated by E_B_ = (E_tot_ − E_sub_ − E_S_ − E_vdW_)/n, where E_tot_, E_sub_, E_S_, and E_vdW_ stand for the electronic energy of the entire system, the system after removing the target atom, the target atom, van der Waals interaction, respectively, and n stands for the number of atoms bonded to the target atom. Here, the van der Waals interaction energy between the target atom and adjacent layer was calculated by E_vdW_ = (E_S+AL_ − E_AL_ − E_S_), where, E_S+AL_ and E_AL_ stand for the electronic energy of the system (adjacent layer and the target atom are held together) and the energy of the adjacent layer, respectively.

### Characteriation

Graphene surface was examined with Raman spectroscopy (WITEC Alpha 300 M^+^) at a wavelength of 532 nm and atomic force microscopy (AFM, Dimension 3100, Veeco). Optical transmittance of the graphene layers was measured by Ultraviolet-visible-near infrared (UV-Vis-NIR) absorption spectroscopy. For optical trasmittance, the trilayer graphene was deposited on glass substrates.

## Results and Discussion

### Ion energy distribution

**Figure 2:**
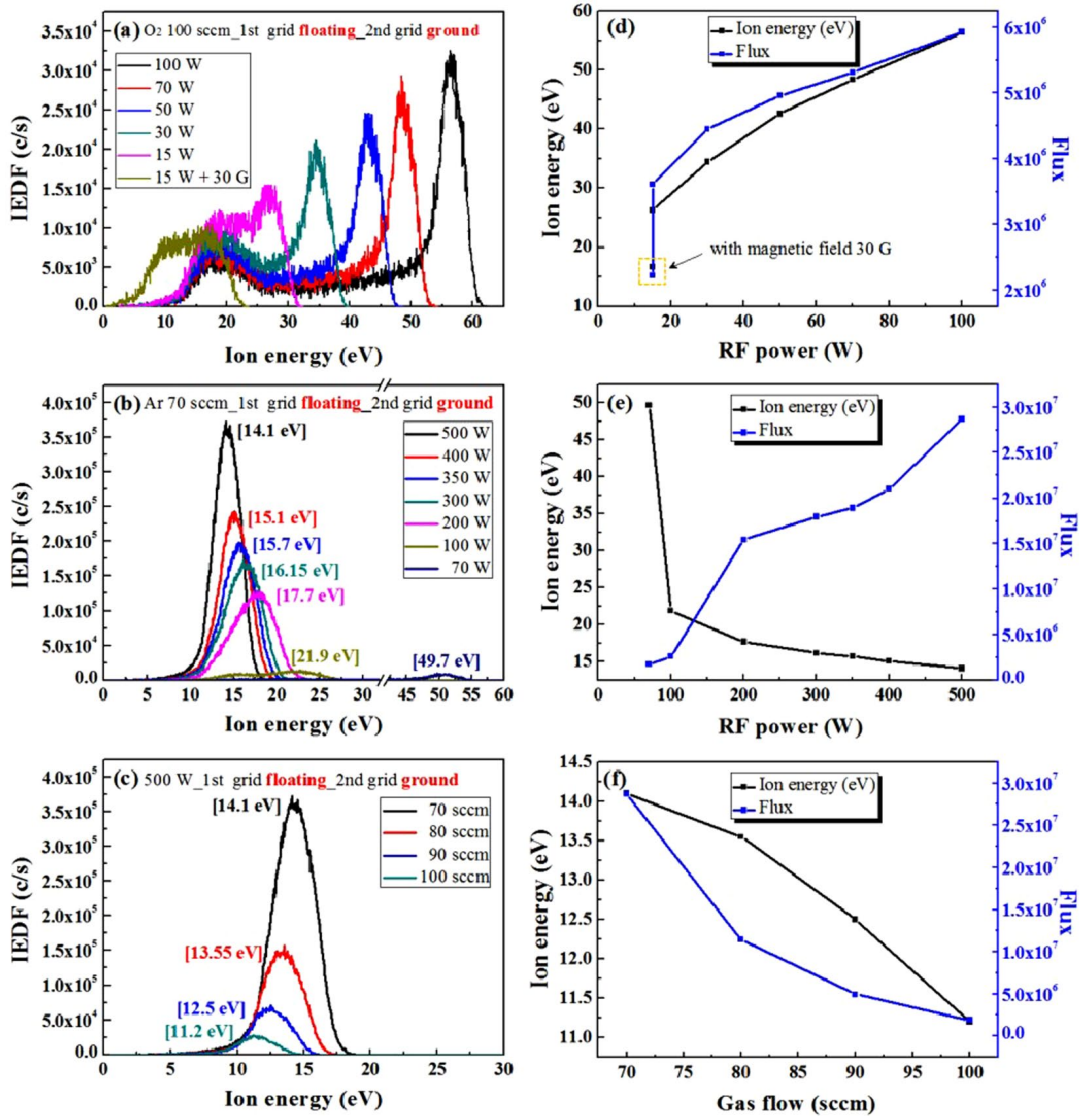
O_2_^+^-ion/Ar^+^-ion energy distribution. Ion energy distribution of the ion source for (**a**) different rf powers at 100 sccm of O_2_ gas flow rate, (**b**) different rf powers at 70 sccm of Ar gas flow rate, and (**c**) different Ar flow rates at 500 W of rf power measured by an ion energy analyzer in the QMS. (**d**,**e** and **f**) are the peak ion energies and fluxes for their O_2_^+^-ion/Ar^+^-ion energy distributions.

Figure 2 shows the ion energies and fluxes of the O_2_^+^-(the intensity of O^+^ ion was much smaller than that of O_2_^+^ as shown in Figure S2, Supplementary Information, therefore, we used the O_2_^+^ only for the oxygen-ion energy analysis) and Ar^+^-ions extracted from the ion source for the operation of sequential steps of ALE composed of chemical adsorption by O_2_^+^-ion and physical desorption by Ar^+^-ion. The 1st and 2 nd grid voltages of the ion gun were floated and grouned, respectively, for low ion energy operation and the rf power or gas flow rates were varied. Figure 2a–c show the O_2_^+^-ion energy distribution measured for different rf powers at 100 sccm of O_2_ gas flow rate, the Ar^+^-ion energy distribution for different rf powers at 70 sccm of Ar gas flow rate, and the Ar^+^-ion energy distribution for different Ar flow rates at 500 W of rf power, respectively. As shown in Fig. 2a, the O_2_^+^-ion energy was spread over a wide range with dual peaks and the decrease of rf power from 100 to 15 W decreased the ion energy. When an axial magnetic field of 30 G was applied to the ion source during the operation at 15 W, the energy was further decreased while having the energy distribution in the range from 0 to 20 eV. Figure 2d shows the peak ion energy and flux of the O_2_^+^-ions in Fig. 2a. The ion flux was increased with the increase of peak ion energy. The characteristics of O_2_^+^-ion energy distribution showing dual ion energy peaks, the increase of ion energy and flux with the rf power suggest the characteristics of capactively coupled plasma (CCP) for oxygen plasma not ICP possibly due to low electron density for oxygen plasmas. To use a low energy oxygen-ion for chemical adsorption step, we used the condition of 15 W of rf power with 30 G of magnetic field at 100 sccm of O_2_ gas flow rate. In the case of Ar^+^-ion, as shown in Fig. 2b, the ion energy distribution was a gaussian type with one peak and the increase of rf power from 70 to 500 W decreased the peak ion energy from 49.7 to 14.1 eV. As shown in Fig. 2e, in the case of Ar^+^-ion, the increase of rf power decreased ion flux to the substrate. The differences between Fig. 2a,b,d,e are believed to be originated from the different operation modes of the ion source, where, an ICP mode is operating for the Ar plasma due to higher electron density while a CCP mode is operating for the oxygen plasma due to lower electron density because oxygen has a high electron affinity while Ar has no electron affinity. When Ar gas flow rate was increasd from 70 to 100 sccm during the operation of the ion source at 500 W, as shown in Fig. 2c, the ion energy was further decreased from 14.1 to 11.2 eV even though the flux to the substrate was decreased together as shown in Fig. 2f. Not to damage the graphene surface during the physical desorption step of ALE, a lower Ar^+^-ion energy may be required but, for the physical desorption of chemisorbed species, a sufficient Ar^+^-ion energy is required. Therefore, to investigate the optimized Ar energy condition for the phyiscal desorption of graphene ALE, we etched the graphene surface with the condition in Fig. 2c, that is, with the Ar^+^-ion energy in the range from 11.2 to 14.1 eV.

### Raman spectra of monolayer grapehene at various process conditions

**Figure 3:**
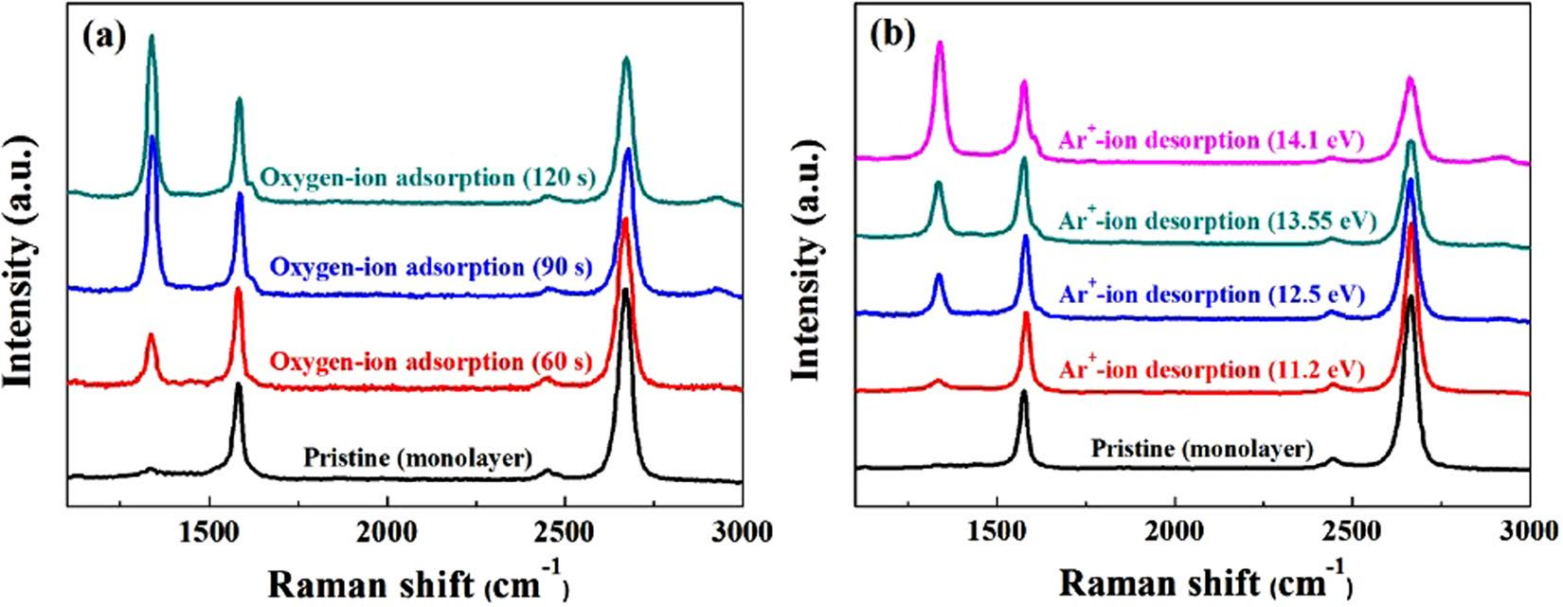
Raman spectra of monolayer graphene to different oxygen-ion adsorption time and Ar^+^-ion energies. (**a**) Monolayer graphene after exposure to an optimize the oxygen-ion condition for different chemical adsorption time and (**b**) monolayer graphene after exposure to different Ar^+^-ion energies. For comparision, Raman spectrum of pristine monolayer graphene was included.

Figure 3a shows the Raman spectra of the monolayer graphene after exposure for different time to an optimized chemical adsorption condition of 15 W of rf power with 30 G of magnetic field and at 100 sccm of O_2_ gas flow rate. For comparison, the Raman spectrum of a pristine monolayer graphene was included. The Raman spectra were normalized by G peak at 1580 cm^−1^ to compare the intensity of D peak and 2D peak accurately. As shown in Fig. 3a, the increase of chemisorption time from 0 to 90 s increased the defect (D) peak at about 1335 cm^−1^ and decreased the 2D peak at about 2675 cm^−1^, however, the further increase of adsorption time to 120 s did not change the peak intensities. The increase of D peak and the decrease of 2D peak with the increase of oxygen-ion adsorption time is believed to related to the oxygen chemisorption on the monolayer graphene network and no further change of D and 2D peak intensities after 90 s suggests the saturation of oxygen chemisorption on the monolayer graphene surface. No change of 2D peak intensity after 90 s also reveals the existence of stable graphene structure up to exposure to 120 s^26, 27^. Figure 3b shows the Raman spectra of the monolayer graphene after exposure to different Ar^+^-ion energies from 11.2 to 14.1 eV in Fig. 2c. Because the ion flux was varied with Ar^+^-ion energy as shown in Fig. 2f, the similar dose was maintained by decreasing the exposure time at high energies; that is, 120 s for 11.2 eV, 46 s for 12.5 eV, 20 s for 13.55 eV, and 8 s for 14.1 eV. As shown in Fig. 3b, for the exposure of monolayer graphene to the 11.2 eV Ar^+^-ion, very small increase or no significant change (because some pristine graphene samples also contain a small D peak) of D peak was observed while the increase of Ar^+^-ion energy signficantly increased the D peak intensity while decreasing the 2D peak intensity indicating significant damage to the graphene surface with increasing the Ar^+^-ion energy. As the results of Fig. 3a,b, for graphene ALE, the chemical exposure time of O_2_^+^-ion was optimized at 90 s and the Ar^+^-ion desorption energy at 11.2 eV.

### Raman spectra of monolayer graphene by optimized one ALE cycle

**Figure 4:**
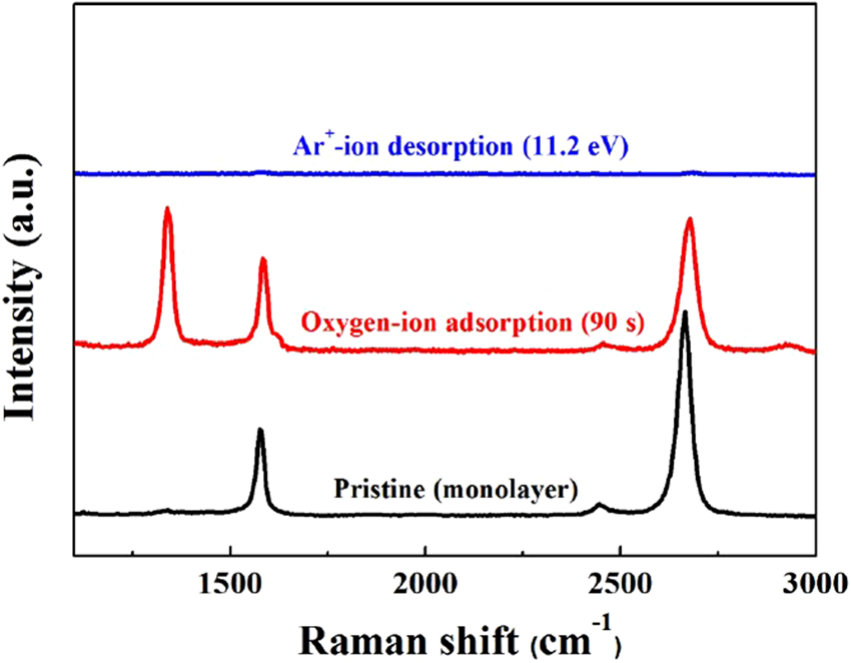
Raman spectra of pristine monolayer graphene by optimized oxygen-ion adsorption step and Ar^+^-ion desorption step. The monol ayer graphene chemically adsorbed with the optimized oxygen-ion for 90 s, and the chemisorbed monolayer graphene sequentially exposed to the optimized Ar^+^-ion for physical desorption for 120 s and which resulted in complete etching of one monolayer graphene.

A pristine monolayer graphene was sequentially exposed using the optimized conditions of the chemical adsorption using oxygen (O_2_^+^/O^+^)-ion (15 W with axial magnetic field of 30 Gauss and 100 sccm of O_2_ for 90 s) and the physical desorption using Ar^+^-ion (500 W and 100 sccm of Ar for 120 s) and the results are shown in Fig. 4. Through one cyle of ALE composed of a chemical adsorption step exposing to the oxygen-ion beam and a sequantial physical desorption step exposing to the Ar^+^-ion beam, one monolayer graphene could be completely removed indicating successful ALE process for graphene.

### Raman spectroscopic data of bilayer graphene during each step of one ALE cycle

**Figure 5:**
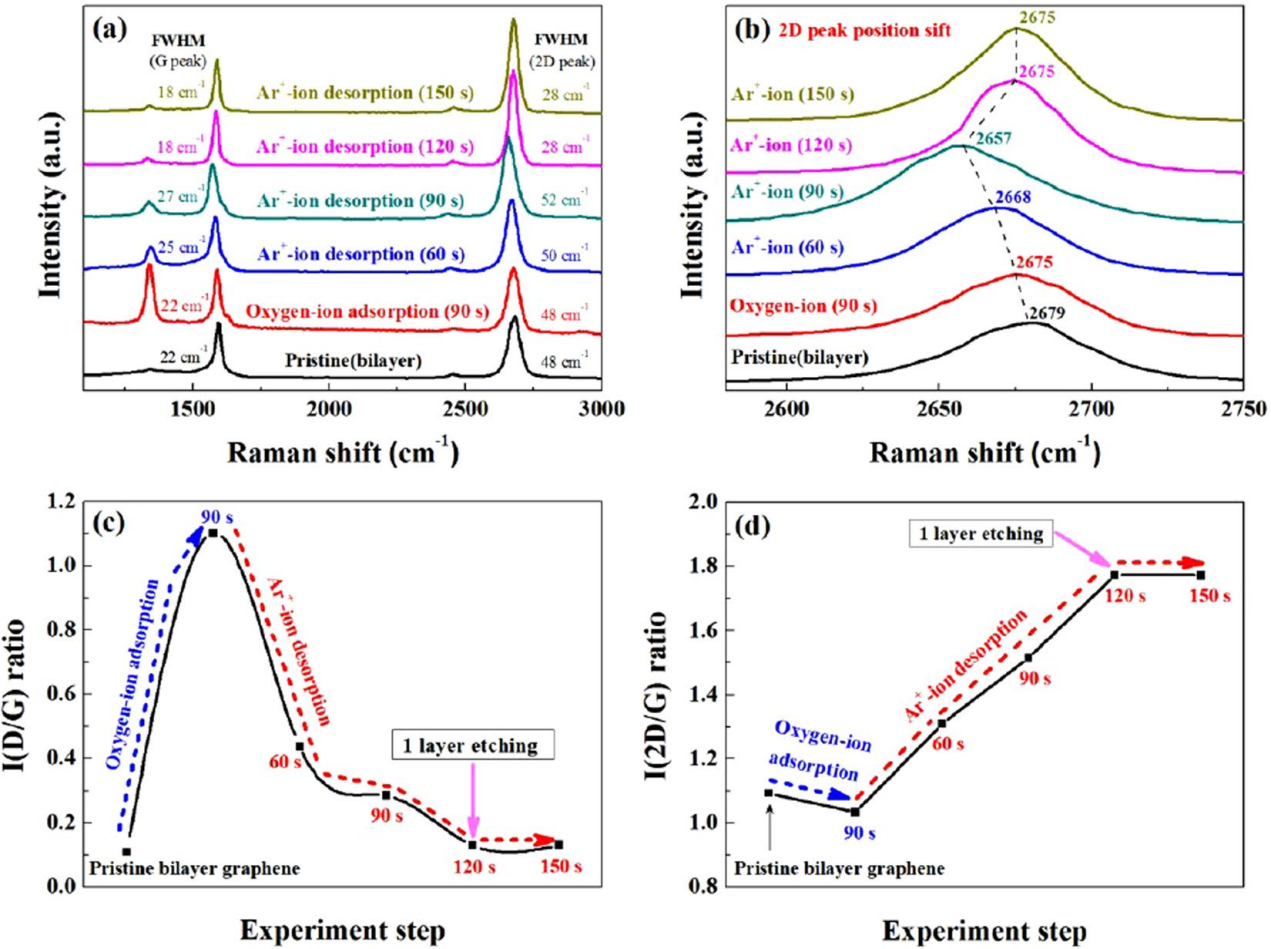
Raman spectroscopic data of bilayer graphene after each step in one cycle of graphene ALE. (**a**) Raman spectra of bilayer graphene after each step in one cycle of graphene ALE. Different time of Ar^+^-ion desorption was conducted after the chemical adsorption of the oxygen (O_2_^+^/O^+^)-ion for 90 s. (**b**) The shift of 2D peak of Raman spectra after oxygen-ion adsorption and after the following Ar^+^-ion desorption for different time. (**c** and **d**) are the change of the peak intensity ratio of D/G and 2D/G of the bilayer graphene Raman spectra in (**a**), respectively.

Using a pristine bilayer graphene, one cycle of graphene ALE was processed and the Raman data of the processed bilayer graphene during each step of one ALE cycle are shown in Fig. 5a. Raman spectrum of the pristine bilayer graphene is also shown. As shown, after 90 s expsoure to the the optimized oxygen-ion for chemical adsorption, the D peak intensity was increased while the 2D peak intensity was decreased as before. After that, the chemisorbed bilayer graphene was exposed to the optimized Ar^+^-ion for 60, 90, 120 (one monolayer etch condition), and 150 s (overetch condition). As shown in Fig. 5a, with increasing the Ar^+^-ion exposure time to 120 s, the D peak intensity was decreased significantly near to zero due to the removal of the top chemically modified graphene layer on the bilayer graphene while increasing the 2D peak intensity due to the formation of monolayer graphene from bilayer graphene. The increase of Ar^+^-ion desorption time further to 150 s after the removal of one monolayer graphene on bilayer graphene did not change the Raman peak intensities indicating no damage or change of graphene structure exposed after the ALE.

The shift of the 2D peak position for the bilayer graphene was investigated after the oxygen-ion chemisorption for 90 s and after the following Ar^+^-ion exposure time from 60 to 150 s, and the results are shown in Fig. 5b. For the prisitine bilayer graphene, the 2D peak was located at 2679 cm^−1^, however, after the 90 s exposure to the oxygen-ion during the chemisorption step, the peak position was red shifted to 2675 cm^−1^. The exposure to the Ar^+^-ion for the desorption of C-O chemisorbed species to 90 s further red shifted the 2D peak to 2668 and 2657 cm^−1^ for 60 s and 90 s, respectively. The red shift of 2D peak after the exposure to the oxygen-ion and Ar^+^-ion is believed to be related to the strain of C-C bonding in the low binding energy C-C structure of the top graphene layer by the C-O bonding and Ar ion bombardment^28, 29^. However, as the top graphene layer is removed and as the second graphene layer is exposed by the Ar^+^-ion exposure for 120 s, the 2D peak position was recovered similar to that of the pristine bilayer graphene. The 2D peak position after Ar^+^-ion exposure of 120 s was about 4 cm^−1^ lower than that of pristine bilayer graphene because the graphene layer changed from a bilayer to monolayer as seen in Fig. 5a ^29, 30^. When the Ar^+^-ion exposure time was further increased to 150 s, no change of 2D peak position was also observed. In addition, as the graphene layer is decreased from bilayer to monolayer by the Ar^+^-ion exposure, the full width at half maximum (FWHM) of the G and 2D peaks was decreased after the formation of monolayer graphene after the Ar^+^-ion exposure of 120 s and the further increase of the exposure time to 150 s did not change the FWHM, either. The FWHM of the G and 2D peaks of pristine bilayer graphene after one cycle ALE decreased from 22 to 18 cm^−1^ and from 48 to 28 cm^−1^, respectively (as shown in Fig. 5a). The FWHM values according to the number of graphene layers are similar to those of the previous studies^26, 27, 31^.

Figure 5c,d show the peak intensity ratios of D/G and 2D/G, respectively, measured from the peak intensity data in Fig. 5a. The intensity ratio of D/G, that is, I(D/G) in Fig. 5c not only shows the degree of defect in the graphene structure but also shows the degree of chemisorption of graphene layer by oxygen-ion. The I(D/G) was increased from 0.11 (pristine) to 1.102 by the chemical adsorption of oxygen-ion on the graphene surface, however, after the Ar^+^-ion exposure of 60, 90, and 120 s, the I(D/G) was decreased to 0.437, 0.285, and 0.130, respectively. Especially, the I(D/G) for the Ar^+^-ion exposure of 120 s was similar to that of the pristine graphene indicating the removal of chemically modified top graphene layer. Even after Ar^+^-ion exposure for 150 s, I(D/G) was 0.131, indicating no change in D peak (that is, no increase of damage) by the overexposure by Ar^+^-ion possibly due to a low energy Ar^+^-ion beam (11.2 eV). In the case of I(2D/G) in Fig. 5d, it shows the number of graphene layers in the graphene sample. As shown, when the pristine bilayer graphene was adsorbed by oxygen-ion during the chemisorption step, the I(2D/G) was changed from 1.094 (pristine bilayer) to 1.035, therefore, no significant change was observed signifying the maintenance of bilayer graphene even after the chemisorption by oxygen-ion. However, after the exposure to the Ar^+^-ion for 60, 90, and 120 s, the I(2D/G) was increased to 1.310, 1.515, and 1.773 showing the formation of monolayer graphene by the removal of top graphene layer^26, 27^. In addition, I(2D/G) after Ar^+^-ion desorption further to 150 s did not change the I(2D/G) which shows that the ALE process (that is, one monolayer removal) was completed at Ar^+^-ion exposure time of 120 s.

The layer-by-layer removal of graphene by the optimized ALE cyclic steps (for chemical adsorption step, an oxygen-ion beam generated with 100 sccm of O_2_, 15 W of rf power, and with axial magnetic field of 30 Gauss for 90 s and, for the physical desorption step, an Ar^+^-ion beam generated with 100 sccm of Ar and 500 W of rf power for 120 s) could also confirmed by etching trilayer graphene and, by observing the change of the optical transmittance at 550 nm. After one, two, and three cycles of ALE using trilayer graphene, the optical transmittance of the etched graphene at 550 nm increased to 94.7, 97.2, and 99.7%, respectively, indicating the formation of bilayer, monolayer, and no layer, therefore, one monolayer removal for one cycle ALE could be confirmed (Figure S3, Supplementary Inforamtion). The selective removal of the chemisorbed top graphene layer only for a bilayer graphene by a low energy ion bombardment could be also expected by the simulation which showed the significant decrease of carbon binding energy in the top graphene layer by the oxygen-ion adsorption while no significant change of binding energy was measured for the carbon bonding in the bottom graphene layer (Figure S4, Supplementary Information).

### Surface morphologies and Raman spectroscopic data of the same sample before and after one cycle ALE

**Figure 6:**
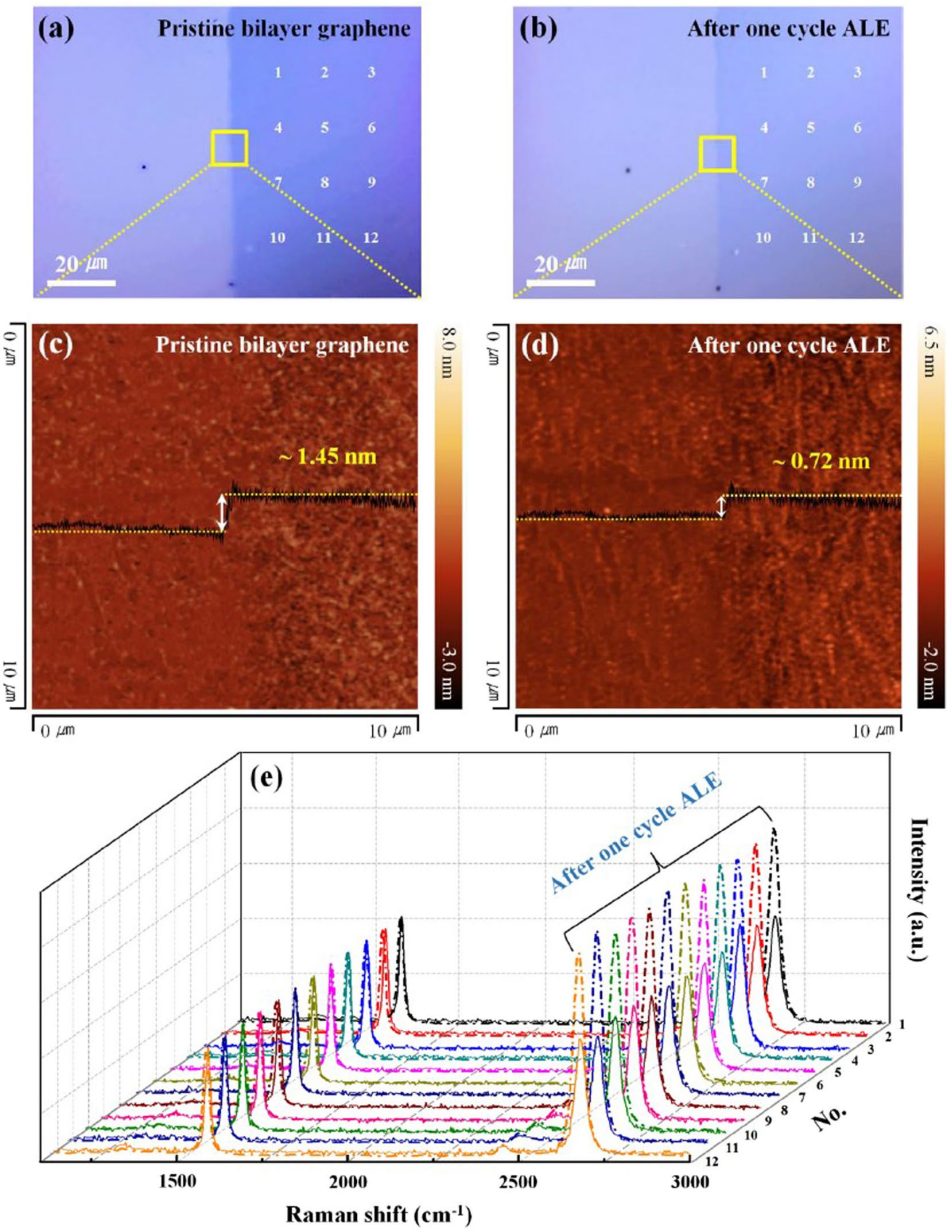
Comparision of bilayer graphene at the same position after one cycle ALE. (**a** and **b**) Optical images of the pristine bilayer graphene before and after one cycle ALE, respectively. (**c** and **d**) AFM images of the same sample before and after one cycle ALE, respectively, for the position denoted by the yelloew square in (**a**) and (**b**). (**e**) Change of the Raman spectroscopic data of the pristine bilayer graphene after one cycle ALE for the positions of 1~12 in (**a** and **b**).

Figure 6 shows the surface morphologies and Raman spectroscopic data of the same sample before and after one cycle ALE of the pristine bilayer graphene. Figure 6a,b are the optical images showing the changes after one cycle ALE. Figure 6c,d show that the thickness of pristine bilayer graphene decreased from ~1.45 to ~0.72 nm after one cycle ALE by AFM images for the position denoted by the yellow square in Fig. 6a,b. At the same time, the rms roughness values before and after one cycle ALE showed ~0.41 and ~0.43 nm, respectively, indicating that one graphene layer was uniformly removed. Figure 6e shows Raman spectroscopic data of pristine bilayer graphene before and after one cycle ALE for the positions denoted by 1~12 in Fig. 6a,b. The average I(D/G) was almost unchanged from ~0.11 to ~0.13 after one cycle ALE in the pristine bilayer graphene, and the average I(2D/G) was increased from ~1.08 to ~1.78. At the same time, the average FWHM of the G and 2D peaks decreased from ~48.5 to ~27.8 cm^−1^ and from ~22.4 to ~17.6 cm^−1^, respectively, and the G and 2D peaks were red shifted from 2679 to 2675 cm^−1^. These results show that the atomic layer etching technique can remove graphene one layer uniformly over a large area without causing noticeable damage to the graphene surface.

## Conclusions

To precisely control the graphene layers without inducing damage, we introduced a graphene ALE process, a cyclic etch process composed of chemical adsorption by a low energy oxygen (O_2_^+^/O^+^)-ion (0~20 eV) and followed physical desorption of the chemisorbed species by a low energy Ar^+^-ion beam (11.2 eV). By using the optimized chemical adsorption of oxygen-ion on the pristine graphene surface and the optimized physical desorption using Ar^+^-ion for the graphene ALE process, exactly one monolayer of graphene could be removed per one etch cycle without damaging the graphene surface exposed after the ALE process. Therefore, the graphene layers of the multilayer graphene could be precisely controlled by the number of ALE cycles. One monolayer graphene removal by one graphene ALE cycle could be confirmed not only by the Raman data (after one cyle ALE of bilayer graphene, the intensity ratio of 2D/G increased from ~1.1 to ~1.8 indicating the formation of monolayer graphene after one cycle of ALE of bilayer graphene) but also by measuring optical transmittance of the etched graphene layers (decrease of 2.5% of optical transmittance at 550 nm per one cycle ALE). The comparison of AFM images and Raman spectroscopic data of the bilayer graphene before and after the ALE showed uniform thickness control without noticeable damage on the etched graphene surface. We believe that this ALE technique can be very useful not only for next generation graphene-based nanoelectronic devices but also for other nanoelectronic devices based on 2D materials requring the precise layer control.

## Electronic supplementary material

Figure S1, Figure S2, Figure S3, Figure S4
