# Supplementary material for: Atomic layer etching of graphene through controlled ion beam for graphene-based electronics
Source: Sci Rep. 2017 May 26;7:2462. doi: 10.1038/s41598-017-02430-8 (PMC5446397; doi:10.1038/s41598-017-02430-8)
Supplement: Supplementary file 1 — Figure S1, Figure S2, Figure S3, Figure S4 [file 41598_2017_2430_MOESM1_ESM.doc]

Supplementary Information

Atomic layer etching of graphene through controlled ion beam for graphene-based electronics

Ki Seok Kim ^1^, You Jin Ji ^1^, Yeonsig Nam ^3^, Ki Hyun Kim ^1^, Eric Singh ^1,4^, Jin Yong Lee ^3^, and Geun Young Yeom ^1,2^,*

*^1^. School of Advanced Materials Science and Engineering, Sungkyunkwan University, 2066 Seobu-ro, Jangan-gu, Suwon-si, Gyeonggi-do 16419, Republic of Korea.*

*^2^. SKKU Advanced Institute of Nano Technology (SAINT), Sungkyunkwan University, 2066 Seobu-ro, Jangan-gu, Suwon-si, Gyeonggi-do 16419, Republic of Korea.*

*^3^. School of Chemistry, Sungkyunkwan University, 2066 Seobu-ro, Jangan-gu, Suwon-si, Gyeonggi-do 16419, Republic of Korea.*

*^4^. Department of Computer Science, Stanford University, Stanford, California 94305, United States*

*Corresponding Author : gyyeom@skku.edu

**The unit cells of the graphene bilayers for AA-stacking and AB-stacking**


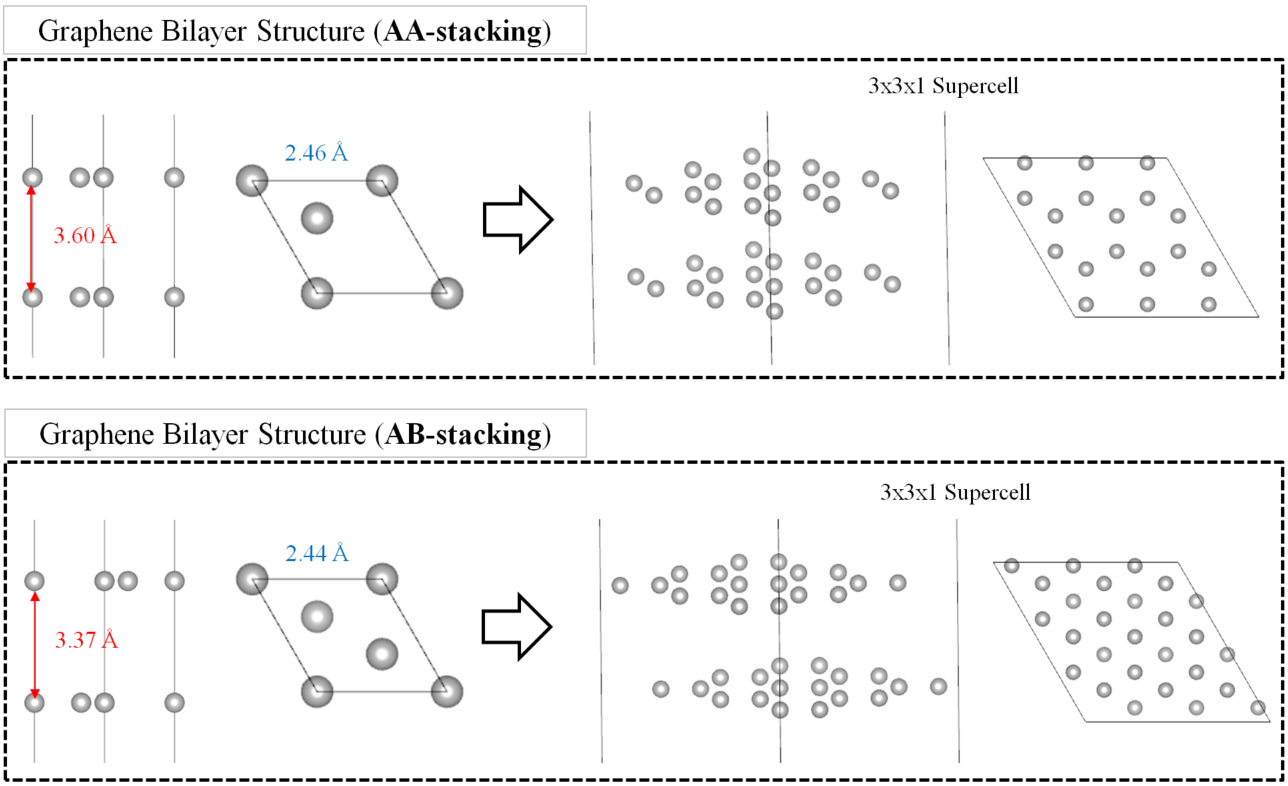


**Figure S1.** The unit cells of graphene bilayers having AA-stacking and AB-stacking used in the simulation.

The calculated lattice constants of the graphene bilayers for AA-stacking and AB-stacking were 2.46 and 2.44 Å, respectively, and the calculated distances between the two graphene layers in the bilayer graphene were 3.60 and 3.37 Å, repsectively. These calculated values were the same as the values studied by other researchers^1,2^. Using the 3x3x1 supercell, the structure of the unit cell was optimized and the right structures in the figures were obtained for both AA-stacking and AB-stacking.

**Oxygen ion (O_2_^+^/O^+^) mass spectra**


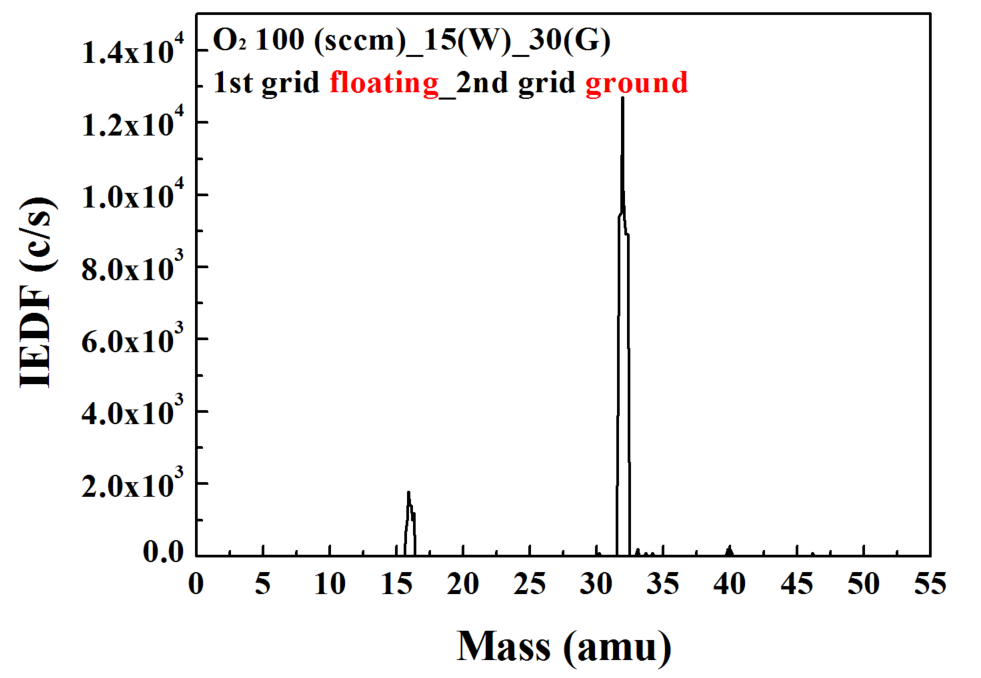


**Figure S2.** Oxygen ion mass spectra obtained at the substrate location during the operation of oxygen ion beam for chemical adsorption.

Oxygen ion mass spectra were measured at the 15 W rf power with 30 Gauss of magnetic field and at 100 sccm of O_2_ gas flow rate. As shown in the figure, as the oxygen positive ion peak, O_2_^+^ and O^+^ were observed and the intensity of O_2_^+^ was much lager than that of O^+^.

**The optical transmittance by the number of ALE cycles**


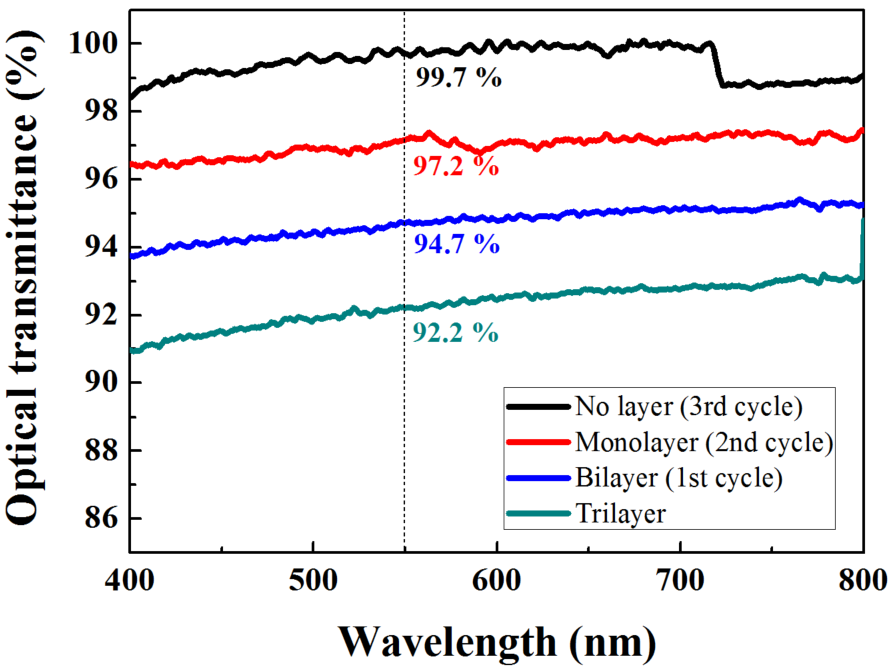


**Figure S3.** Optical transmittance of mono-, bi-, and tri-layer graphene layers obtained by ALE using a trilayer graphene.

Figure shows the optical transmittances of mono-, bi-, and tri-layer graphene layers including no layer obtained by ALE using a trilayer graphene. For optical trasmittance, the trilayer graphene deposited on glass susbstrate was etched by ALE for one, two, and three cycles to obtain bi- and mono-layer graphenes and to etch away the trilaye graphene (no layer graphene). The optical transmittances were measured to indentify the number of graphene layers after one and two cyles of ALE of trilayer graphene. The optical transmittance of the pristine trilayer graphene at 550 nm was about 92.2% and it was close to the theoretical value because one monolayer graphene decreases the optical transmittance at 550 nm about 2.5%^3,4^. After one, two, and three cycles of ALE using trilayer graphene, the optical transmittance of the etched graphene at 550 nm increased to 94.7, 97.2, and 99.7 %, respectively, indicating the formation of bilayer, monolayer, and no layer, therefore, one monolayer removal for one cycle ALE could be confirmed.

**Binding energy changes in AA-stacking and AB-stacking of the bilayer graphene before and after oxygen adsorption**


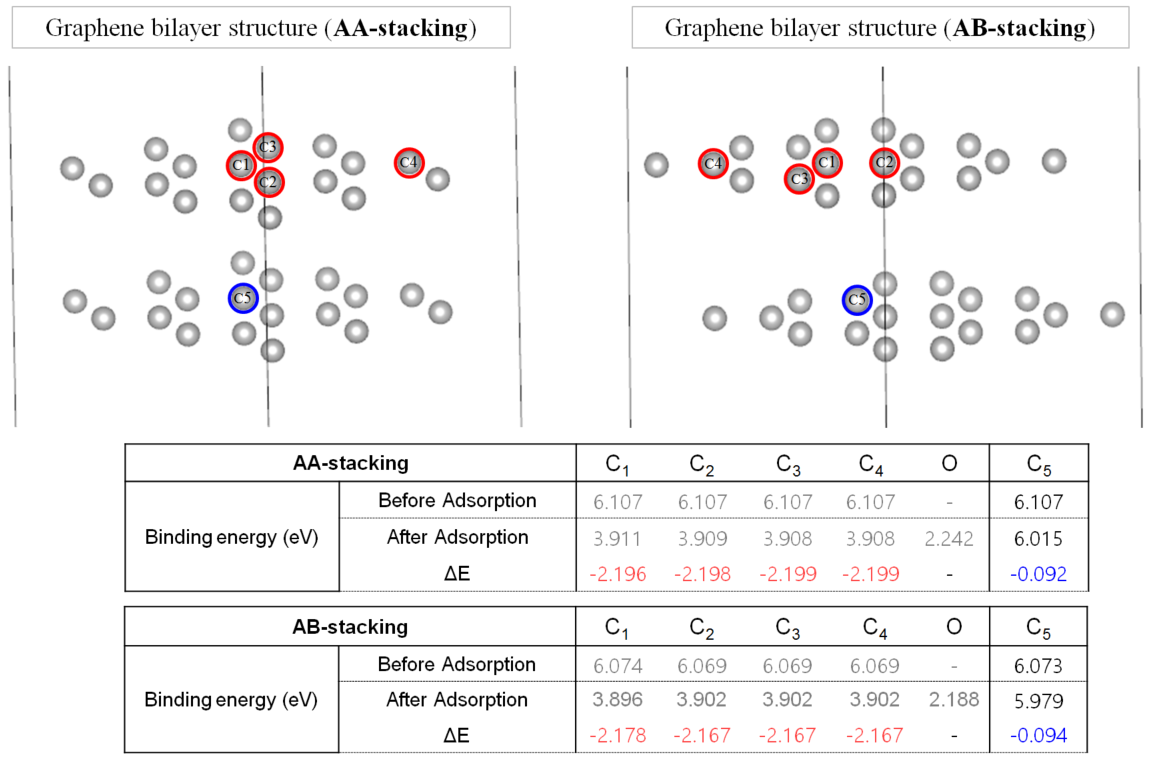


**Figure S4.** The change of carbon binding energies after oxygen adsorption in the bilayer graphenes having AA-stacking structure and AB-stacking structure.

The change of binding energy of the carbons in the top graphene layer of the bilayer graphene with both AA-stacking and AB-stacking after the oxygen adsorption can be obtained from the binding energies of C_1_ ~ C_4_ atoms (red circle) in the top layer of the figure. The change of binding energy of the carbons in the bottom graphene layer of the bilayer graphene after the oxygen adsorption was also investigated and can be understood from the binding energy of C_5_ atom (blue circle) under the C_1_ atom. As shown in the tables under the figures, after the oxygen adsorption, the binding energies of C_1_ ~ C_4_ atoms changed from ~ 6.1 to ~ 3.9 eV for both AA-stacking and AB-stacking, therefore, the binding energy of ~ 2.2 eV was decreased for both AA-stacking and AB-stacking. The decrease of carbon binding energy in the graphene layer is due to the electron migration from carbon to oxygen due to higher electronegativity of oxygen atoms. On the contrary, after the oxygen adsorption on the top graphene layer, the binding energy of C_5_ atom in the bottom graphene of bilayer graphene changed only a little amount of ~ 0.1 eV. Therefore, by using a controlled Ar^+^-ion energy for ALE, only the top graphene layer can be selectively removed without etching and damaging the bottom graphene layer for the bilayer graphene.

**References**

1. Mapasha, R. E., Ukpong, A. M. & Chetty, N. Ab initio studies of hydrogen adatoms on bilayer graphene. *Phys. Rev. B* **85**, 205402 (2012).
2. Hargrove, J., Shashikala, H. M., Guerrido, L., Ravi, N. & Wang, X. Band gap opening in methane intercalated graphene. *Nanoscale* **4**, 4443-4446 (2012).
3. Nair, R. R. *et al*. Fine structure constant defines visual transparency of graphene. *Science* **320**, 1308 (2008).
4. Sun, Z. *et al*. Growth of graphene from solid carbon sources. *Nature* **468**, 549-552 (2010).
